# Green synthesis of silver nanoparticles using cocoon extract of *Bombyx mori* L.: therapeutic potential in antibacterial, antioxidant, anti-inflammatory, and anti-tumor applications

**DOI:** 10.1186/s12896-025-00971-9

**Published:** 2025-05-14

**Authors:** Ahmed H. Elsaffany, Ahmed E. M. Abdelaziz, Abdullah A. Zahra, Alsayed E. Mekky

**Affiliations:** 1 Department of Plant Protection, Faculty of Agriculture, Al-Azhar University, Cairo, Egypt; 2 Department of Botany and Microbiology, Faculty of Science, Port-Said University, 23 December Street, P.O. Box 42522, Port-Said, Egypt; 3 Department of Botany and Microbiology, Faculty of Science, Al-Azhar University, Cairo, 11884 Egypt

**Keywords:** *Bombyx mori*, Silk, Silver nanoparticles, Antioxidant, Anti-inflammatory, Cytotoxic properties

## Abstract

*Bombyx mori* silk is one of the most extensively studied types of silk due to its unique mechanical properties and biocompatibility, which have enabled its Utilization in medical applications Including surgical sutures since the second century. In the present study, a new method for the biosynthesis of silver nanoparticles (AgNPs) was explored by applying *Bombyx mori* cocoon extract as a sustainable and eco-friendly biological source. Unlike previous studies that primarily utilized plant or microbial extracts, this approach offers a more efficient alternative due to the unique protein and polyphenol content of silk cocoons, which enhances the stability and biological properties of the biosynthesized nanoparticles. The resulting AgNPs exhibited significant antibacterial, antioxidant, anti-inflammatory, and cytotoxic properties, opening new avenues for their therapeutic applications. This study expands the range of biological materials used in AgNP synthesis and provides deeper insight into how different bioactive components influence their functional properties. In this study, AgNPs were biosynthesized by mechanically processing extracted raw silk material with silver nitrate (AgNO₃). The synthesized nanoparticles were characterized by implementing several physicochemical techniques, including UV-visible spectrophotometry, FTIR, and XRD, and their morphology was examined through Transmission Electron Microscopy (TEM). The obtained AgNPs displayed a distinct absorption peak at 420 nm, with a particle size ranging between 5 and 25 nm, and displayed characteristic FTIR and XRD patterns typical of silver nanoparticles. The biosynthesized AgNPs demonstrated significant antimicrobial activity against *Staphylococcus aureus* (ATCC25923), *Staphylococcus haemolyticus* (ATCC29968), *Escherichia coli* (ATCC8739), and *Klebsiella pneumoniae* (ATCC2146). The antioxidant potential, assessed via the DPPH assay, yielded an IC50 value of 4.94 µg/ml, while the anti-inflammatory effect, evaluated using the membrane stabilization technique, showed an IC50 of 7.14 µg/ml. Additionally, AgNPs exhibited notable cytotoxic properties against Caco-2 and PANC1 cell lines, with IC50 values of 177.24 ± 2.01 µg/ml and 208.15 ± 2.79 µg/ml, respectively. Conversely, their impact on normal HFB-4 cells was minimal, with an IC50 of 582.33 ± 6.37 µg/ml, indicating a favorable safety profile. These observations highlight the multifunctional potential of silk-derived AgNPs, suggesting their applicability in various biomedical fields.

## Introduction

Nanotechnology has revolutionized biomedical research, particularly in the synthesis of nanoparticles with enhanced therapeutic potential [1]. Among these, AgNPs have garnered significant attention due to their broad-spectrum antimicrobial, antioxidant, anti-inflammatory, and anticancer properties [2]. The interest in AgNPs is driven by their distinctive characteristics, such as electronic, magnetic, mechanical, and chemical properties, as well as tunable surface plasmon resonance, a high surface-to-volume ratio, and their involvement in oxidation reactions [3]. AgNPs have shown significant promise across various fields, such as nanomedicine, biosensing, biological coatings, surface-enhanced Raman scattering, electronics, and are particularly noted for their potential antibacterial properties [4]. The bio-based synthesis methods for nanoparticles are gaining increasing preference over traditional methods, as conventional physical and chemical synthesis techniques are often labor-intensive, costly, and involve the use of hazardous chemicals [5]. While green synthesis using biological templates provides a sustainable and eco-friendly alternative, reducing toxicity and enhancing biocompatibility. Various plant extracts, fungi, and bacteria have been explored for AgNP biosynthesis; however, the selection of an optimal biological template remains a crucial factor influencing nanoparticle properties and applications [6].

AgNPs exhibit antibacterial properties through various mechanisms, and host immune responses may enhance these activities. The antimicrobial efficacy of nanoparticles is influenced by factors such as their physicochemical properties, the method of delivery, and the specific pathogen involved [7]. The broad-spectrum antimicrobial action of nanosilver is attributed to its interaction with multiple molecular targets in microorganisms. Additionally, bacterial septicemia is a common disease in silkworms, typically caused by various pathogenic bacteria, including *Bacillus*,* Pseudomonas*,* Streptococcus*, and *Staphylococcus* species [8]. According to Hemlata et al. [9], AgNPs synthesized from *Cucumis prophetarum* leaf extract were found to range in size from 30 to 50 nm. These nanoparticles exhibited bacterial inhibition zones of 11 ± 0.4 mm, 14 ± 0.3 mm, and 18 ± 0.4 mm for *S. aureus*, and 15 ± 0.2 mm, 17 ± 0.5 mm, and 20 ± 0.6 mm for *S. typhi* at concentrations of 20, 50, and 75 µg/mL, respectively [22]. In the early stages of wound infections, common bacterial species such as *P. aeruginosa*,* S. aureus*,* K. pneumoniae*,* Enterococcus faecalis*, and *Acinetobacter baumannii* are frequently involved, with *S. aureus* being the primary pathogen. As the infection progresses, *P. aeruginosa* often colonizes the wound and can lead to sepsis by invading the lymphatic and blood vessels [10]. A recent study on microbial isolation from wounds and the assessment of antimicrobial resistance found that 29.2% of the isolated strains exhibited resistance to six different antibiotics. Among these, S. aureus and P. aeruginosa were the most prevalent, followed by *E. coli*,* K. pneumoniae*,* P. mirabilis*, and *A. baumannii* [11]. Therefore, to promote effective and efficient wound healing, it is crucial to inhibit the growth of pathogenic bacteria at the wound site. Reactive oxygen species (ROS) play a key role in regulating various stages of wound healing, and their low levels are essential to prevent further injury to the tissue [12]. Wound progression is often associated with redox imbalances, which are a result of increased oxidative stress and reduced antioxidant capacity. Thus, wound dressings should possess both antibacterial and antioxidant properties to prevent infections and control excessive ROS production at the wound site [13, 14]. Cancer remains the leading cause of death worldwide and a major public health concern. It is typically defined by the uncontrolled growth and division of cells in any part of the body, which is driven by genetic mutations and harmful environmental factors [15]. Due to their antioxidant and cytotoxic properties, AgNPs promote cytotoxicity by producing an overabundance of ROS, which triggers apoptosis in both cancerous and non-cancerous cells, including those associated with lung, ovarian, and breast cancers [16].

Silkworms, which belong to the Lepidoptera order, are often referred to as the “Queen of textiles” due to their production of valuable silk fibers [8]. *Bombyx mori* L., a significant economic insect, has made substantial contributions to the development of national economies [17]. Silk from *Bombyx mori* is a valuable biomaterial enriched with fibroin, sericin, and polyphenols, which play a crucial role in the green synthesis of nanoparticles [18]. These bioactive compounds not only act as reducing and stabilizing agents but also provide a controlled and reproducible synthesis environment, distinguishing silk-derived biomolecules from plant-based extracts [19–22]. The structural integrity and biocompatibility of silk proteins contribute to the formation of nanoparticles with enhanced stability and bioactivity. In particular, sericin exhibits notable antioxidant and antimicrobial properties, which can improve the therapeutic potential of the synthesized nanoparticles [23]. Additionally, its biodegradability and film-forming capabilities make it a suitable candidate for biomedical applications, including drug delivery, wound healing, and tissue engineering. The ability of sericin to modulate nanoparticle size and shape further underscores its significance in nanotechnology, making silk a promising alternative for sustainable and efficient nanoparticle synthesis [24, 25].

Despite numerous studies on the synthesis of AgNPs from various biological sources, the use of *Bombyx mori* silk cocoons for the biosynthesis of these nanoparticles remains underexplored, making this study a novel step in this field. This study aims to bridge this gap by exploring the biosynthesis of AgNPs using *Bombyx mori* cocoon extract and evaluating their therapeutic potential. By leveraging the unique biochemical composition of silk, this approach seeks to develop highly stable, biocompatible, and multifunctional AgNPs suitable for antimicrobial, antioxidant, anti-inflammatory, and cytotoxic properties.

## Materials and methods

### Materials & rearing of silkworm

**Fig. 1:**
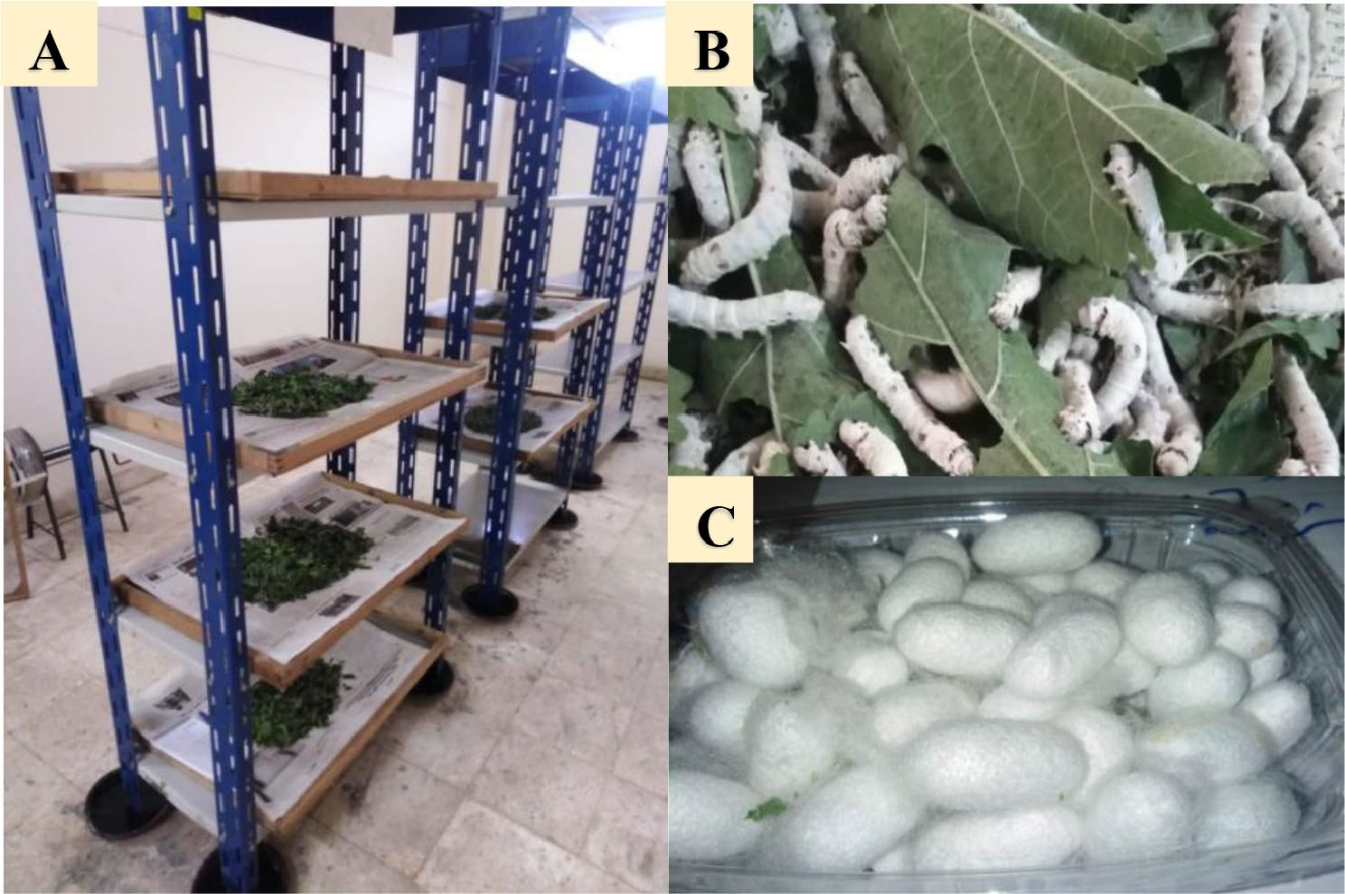
Sericulture of the silk worm (**A**) Rearinig of various groups of silkworm in Silkworm Unit in Faculty of agriculture in Al-Azhar University; (**B**) Feeding of silk worm on Mulberry leaves; (**C**) The produced cocoon

All chemicals and kits used in this study were sourced from Sigma, Egypt. The bacterial strains were generously supplied by the Culture Collection Unit at the National Research Center. **Silkworm hybrid resources**: Eggs of the silkworm hybrid Vra Tza, Bulgaria silkworm (B. mori, L.) were obtained from the Sericulture Research Department of Plant Protection Research Institute, Agricultural Research Centre, Giza- Egypt. Experimental materials: These experiments were conducted in the laboratories of Plant Protection Department., Faculty of Agriculture, AL-Azhar Univ, Nasr City, Cairo, Egypt during spring rearing season, 2023 using leaves of Mulberry Mours indica Kanva-2 planted in its experimental farm as shown in Fig. 1. **Silkworm rearing and cocoons production**: Prior to the commencement of rearing, the room and equipment’s were cleaned, washed and properly disinfected with four per cent formalin by using knapsack sprayer. The room was kept air tight for 48 h for effective disinfection. A day prior to rearing, room was kept open to expel the traces of formaldehyde and then the rearing equipment’s were arranged for silkworm rearing as shown in Fig. 1. **Silkworm Life Cycle Stages**: Eggs (Grainage): Incubate eggs at 24–25 °C and 75–80% humidity in dim light until hatching. Larvae (Rearing) Chawki Rearing (1st & 2nd Instars): Feed young silkworms tender mulberry leaves. Maintain 26–28 °C and 85–90% humidity. Late Rearing (3rd to 5th Instars) Feed coarser leaves at regular intervals. Maintain 23–26 °C and 70–75% humidity. **Moulting**: Silkworms stop feeding during moulting; avoid disturbance and maintain cleanliness. **Feeding Management**: Feed four times daily for optimal growth. Chop leaves finely for early instars; use whole leaves for later instars. **Spacing and Bed Cleaning**: Gradually increase spacing as silkworms grow to prevent overcrowding. Remove leftover leaves and feces regularly using nets. **Mounting for Cocoon Formation**: Transfer mature larvae (golden yellow with reduced activity) to spinning. Ensure a warm and dark environment with 24–26 °C temperature and 70% humidity. **Cocoon Harvesting**: Harvest cocoons 5–7 days after spinning, ensuring pupae have hardened. Sort and grade cocoons based on quality parameters like size, weight, and silk content [26].

### Processing of raw material

The silkworm cocoon was thoroughly cleansed with deionized water to remove surface proteins after being heated twice in a 0.6% (w/v) Na₂CO₃ solution. Once fully dried, the silk was combined with a traditional reagent mixture of water, anhydrous ethanol, and calcium chloride (1:2:8 molar ratio) and heated at 80 °C for two hours. To enhance purity, unwanted materials were removed by centrifugation at a constant temperature for 10 min at 8300× g. A cellulose semipermeable membrane (MWCO = 1400 Da) was employed to eliminate residual calcium chloride and other contaminants from the supernatant by dialysis in deionized water for three days. After filtration and freeze-drying, the final recovered silk fibroin (RSF) was stored at 4 °C [27].

### Preparation of nanoparticles

RSF was dispersed in deionized water at a concentration of 10 mg/mL. Similarly, AgNO₃ was solubilized in deionized water at a concentration of 10 mM. The RSF solution was then vigorously stirred while the AgNO₃ solution was added dropwise. This led to the immediate formation of an emulsion, which was stirred for 30 s and subsequently subjected to ultrasonication at 30% amplitude for 2 min at 0 °C. The mixture was then centrifuged at 15,000× g for 25 min, and the obtained nanoparticles were washed, lyophilized, and stored at -20 °C [28].

### Characterization of AgNPs

The UV-visible absorption spectra of AgNPs were recorded using a UV-3010 spectrophotometer (AGILENT Cary 60, USA). The nanoparticle morphology was examined using transmission electron microscopy (TEM) with a JOEL instrument (Japan). For Fourier transform infrared (FTIR) analysis, the colloidal silver solution was mixed with acetone, and the precipitate was dried before being characterized. FTIR spectra were collected with a Polymer ID FTIR analyzer L160001J (PerkinElmer, USA), covering a spectral range of 400–4000 cm⁻¹ and a resolution of 4 cm⁻¹. X-ray diffraction (XRD) patterns of the silver nanoparticles were obtained using a D/max3c X-ray diffraction system (ThermoFisher, USA) with Cu Kα radiation (λ = 1.5406 Å), scanning at 2°/min over a 2θ range of 20° to 80°, with a step size of 0.02°. Peak identification and phase determination were performed using Match! software and refined with HighScore Plus [29, 30].

### Detection of antibacterial action, minimal inhibitory concentration (MIC) and minimum lethal level (MLC)

The well agar diffusion method was used to evaluate the antimicrobial effect of AgNPs in vitro against a range of test microorganisms. The bacteria were placed on Mueller Hinton agar plates. For the bacteria, a total of 100 µl of different suspended bacterial cells (1.7 × 10^6^ colony forming units/ml) were used. The analyzed specimens were subsequently transferred to the well that had been made in medium using a cork borer edge that had been cleaned. Gentamicin (0.06 mg/ml) was the standard, and DMSO served as the solvent control. The inhibitory zone was assessed five to seven days after the bacterial cells had developed for 72 h at 36 °C [31].

In 96 well micro-titer plates, the micro-dilution broth method was applied for determining the MIC and MLC. For the examined bacteria, the ultimate bacterial suspension levels in the wells were 5–9 × 10^5^ CFU/mL. Prior to the microbial suspensions being injected in each well, the produced nanoparticles were serially diluted in water. For a whole day, the plates underwent incubation at 37 °C. After that, each well received 15 µL of 0.01% Resazurin (Sigma-Aldrich, Egypt) and was incubated for 4 h to encourage oxidation-reduction and assess the vitality of the cells by looking for a shift in color. To find the MLC, the wells that corresponded to the MIC and at least three prior wells were homogenized, diluted six times, and then plated on Mueller-Hinton agar. For twenty-four hours, the plates underwent incubation at 37 °C. The amount of live bacteria was calculated in CFU/mL by counting the colonies. When more than 50–100% of the examined microbial culture had been eliminated by the silver nanoparticles or fractions, MLC was taken into consideration. The positive control was gentamicin. Cultures without antibacterial agents in their media served as negative controls. In three separate trials, assays were conducted three times for every bacterium [32, 33].

### Estimation of antioxidant activity

To evaluate the AgNPs’ antioxidant potential produced. The 0.10 mM DPPH-containing ethanol solution was utilized. One milliliter of various specimen levels in ethanol, ranging from 3.9 to 1000 µg/ml, was combined with three milliliters of this solution. After giving the mixture a good shake, it was let stand for 35 min at the ambient temperature. The color that results from measuring the absorbance at 518 nm [34].

### Determination of anti-inflammatory impact

The purpose of the membrane stabilization test was to ascertain both samples’ anti-inflammatory properties. To do this, many sample concentrations ranging from 100 to 1000 µg/ml were created. Hypotonic liquid was used to incubate the acquired samples. Indomethacin and distilled water were used as positive and negative standards, respectively. 500 µL of the samples were combined with the fresh erythrocyte suspension (3.0%) in 0.8 mL of saline, and the mixture was incubated for two hours at 36 °C. After that, the mixture was spun for 20 min at 6 °C at 13,000 ×g. At 570 nm, the samples’ absorbance was measured [35].

### Estimation of cytotoxic properties

After dissolving the prepared AgNPs in DMSO, the antineoplastic effect on PANC1(Human pancreatic carcinoma cells) and Caco-2 cells (colorectal adenocarcinoma cells) as well as HFB-4 cells (Human fibroblast cells as normal cells) was identified using the MTT technique. The outcome, when standard concentrations were used, resulted in a blue color, with the intensity of the hue correlating directly to the number of viable cells. Absorbance was then measured at 570 nm using a computerized microplate reader (Agilent Bioteck Neo, USA). After a 24-hour attachment period, with samples ranging from 1000 to 31.25 µg/mL in concentration, the cells were maintained at 37 °C for an additional 24 h. Following the addition of fresh medium, 100 µL of MTT solution (5.0 mg/mL) was introduced, and the mixture was incubated for four hours at 35 °C. To observe the cells, a CCD camera was connected to a microscope (Olympus, Japan) [36].

### Statistical assessment

The data are expressed as mean ± standard deviation (SD) for each experiment, performed in triplicate. Statistical analysis was carried out using one-way ANOVA, followed by Tukey’s post hoc test, to assess differences between groups. The analysis was performed using GraphPad Prism V5 (San Diego, CA, USA). A p-value of less than 0.05 was considered statistically significant. The F-value and its associated p-value were reported to represent the extent of variance between groups.

## Results

### Physicochemical characterization of the prepared nanoparticles

**Fig. 2:**
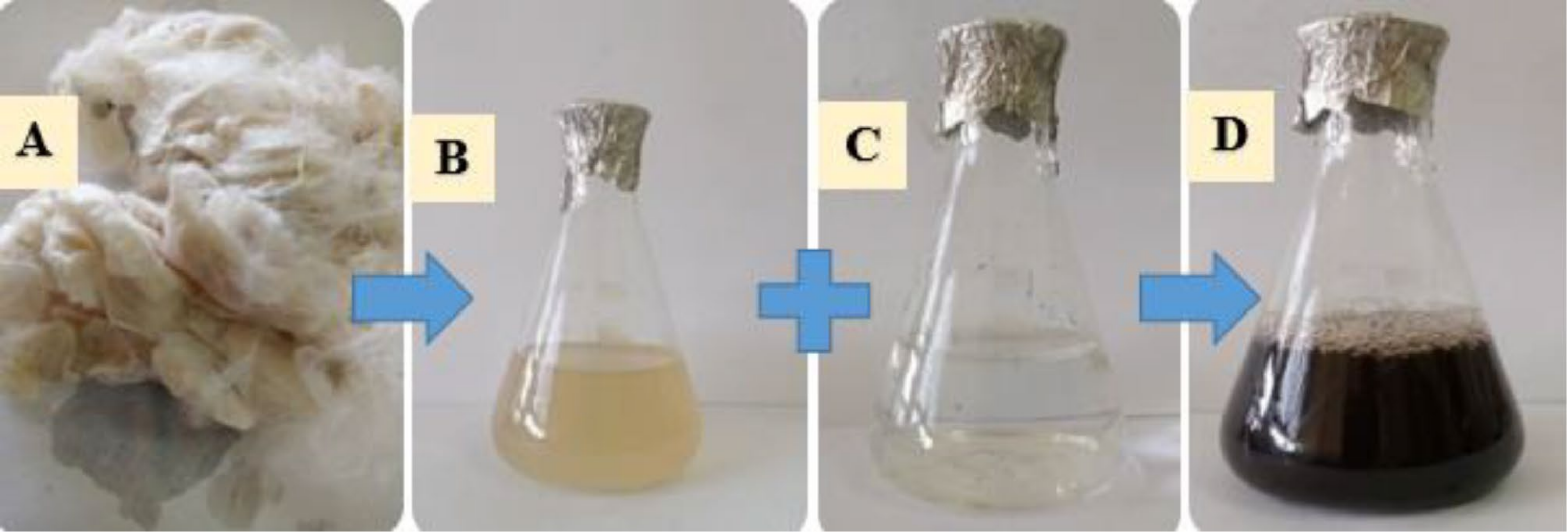
**A**: The used *Bombyx mori* silk; **B**: *Bombyx mori* extract, **C**: Silver Nitrate; **D**: Changing the color after prior to preparation of AgNPs

**Fig. 3:**
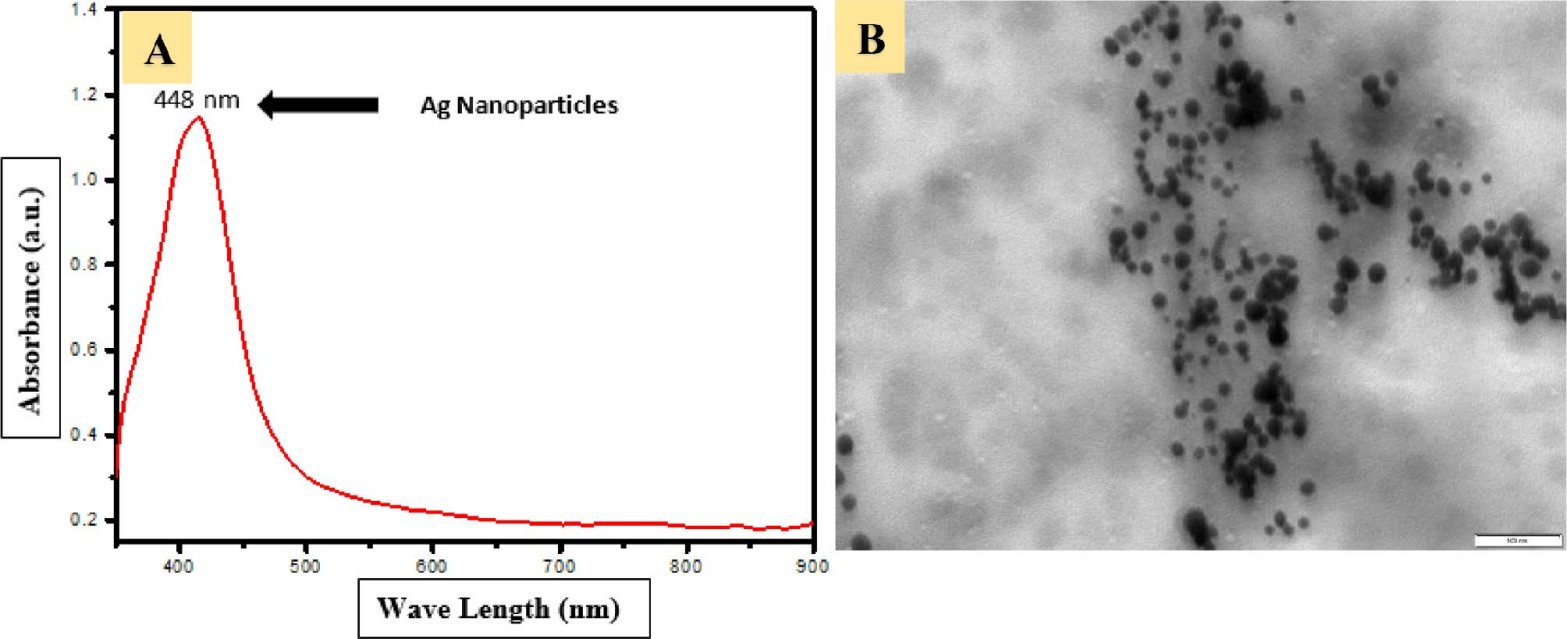
**A**: UV- visible spectrophotometry for the prepared AgNPs with a distinct peak at 420 nm; **B**: TEM micrograph for the prepared AgNPs illustrating their different sizes ranging from 5–25 nm

The prepared solution exhibited a black coloration (Fig. 2D), indicating the successful biosynthesis of AgNPs. To confirm their presence, the solution was analyzed using UV-visible spectroscopy within a wavelength range of 200–900 nm. A distinct surface plasmon resonance (SPR) peak was observed at 420 nm when scanning between 400 and 450 nm, which is characteristic of silver nanocrystals (Fig. 3A). The intensity and position of this peak further validate the formation of stable silver nanoparticles in the colloidal solution.

To investigate the morphology and size distribution of the biosynthesized nanoparticles, TEM analysis was performed. The TEM images (Figs. 3B) confirmed the presence of well-dispersed, spherical silver nanoparticles with a size range of 5–25 nm. The uniformity in particle size suggests controlled synthesis, which is essential for maintaining consistent physicochemical properties.

**Fig. 4:**
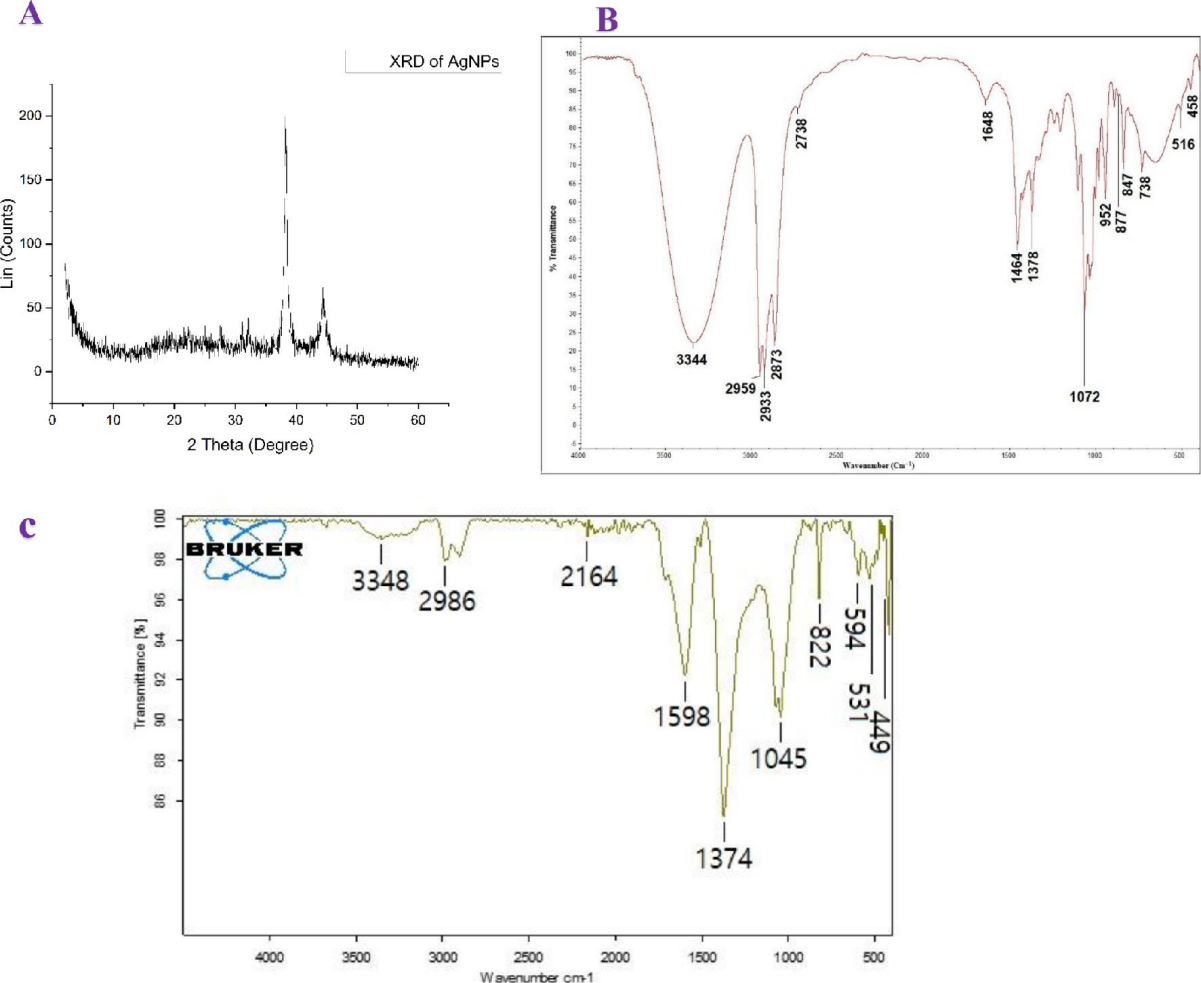
**A**: XRD for the prepared AgNPs with various distinctive peaks; **B**: FTIR of *Bombyx mori* silk; **C**: FTIR for the APNPs prepared by *Bombyx mori* silk

Furthermore, the crystalline structure of the biosynthesized AgNPs was analyzed using XRD. The XRD pattern (Fig. 4A) revealed three distinct diffraction peaks at 2θ values of 32.07°, 38.25°, and 44.41°, corresponding to the characteristic reflections of AgNPs. These peaks confirm the crystalline nature of the nanoparticles. The average crystallite size was calculated using Scherrer’s equation, yielding a mean particle size of approximately 20 nm (Table 1). Notably, this size estimation aligns well with the dimensions observed in the TEM analysis, reinforcing the reliability of the structural characterization.

**Table 1:** Various peaks of silver nanoparticles in X-ray diffraction

Peak number	Pos. [°2Th.]	d-spacing [?]	Height [cps]	Rel. Int. [%]
1	32.0734	2.79069	21.07	9.32
2	38.2572	2.35264	225.96	100.00
3	44.4145	2.03975	50.43	22.32

The FTIR spectrum of *Bombyx mori* silk (Fig. 4B) shows a broad absorption peak at 3344 cm⁻¹, indicating N-H stretching vibrations. Peaks at 2959, 2873, and 2833 cm⁻¹ correspond to C-H stretching vibrations. The strong peak at 1648 cm⁻¹ represents C = O stretching in the amide I region, while peaks at 1464 and 1378 cm⁻¹ correspond to amide II and III bands. Additional peaks at 1072, 877, and 783 cm⁻¹ suggest the presence of C-O and C-H bending vibrations. On the other hand, the FTIR spectrum of AgNPs Prepared Using *Bombyx mori* Silk (Fig. 4C) exhibits a peak at 3348 cm⁻¹, indicating O-H or N-H stretching vibrations. A peak at 2986 cm⁻¹ corresponds to C-H stretching vibrations, while the peak at 2164 cm⁻¹ may be associated with C ≡ C or C ≡ N stretching. The peaks at 1598 and 1374 cm⁻¹ represent C = O and C-N vibrations indicating the presence of proteins that may act as stabilizing agents for AgNPs, while peaks at 1045 suggesting the presence of sugars or proteins contributing to nanoparticle stabilization. The peaks observed at 594 cm⁻¹, 531 cm⁻¹, and 449 cm⁻¹ are associated with metal-oxygen (M-O) vibrations, indicating the successful formation of silver nanoparticles. These results suggest that biomolecules, including phenolic compounds, proteins, and carbohydrates, are integral to the green synthesis and stabilization of AgNPs.

### Antibacterial impact & MIC and MLC of the prepared AgNPs

**Fig. 5:**
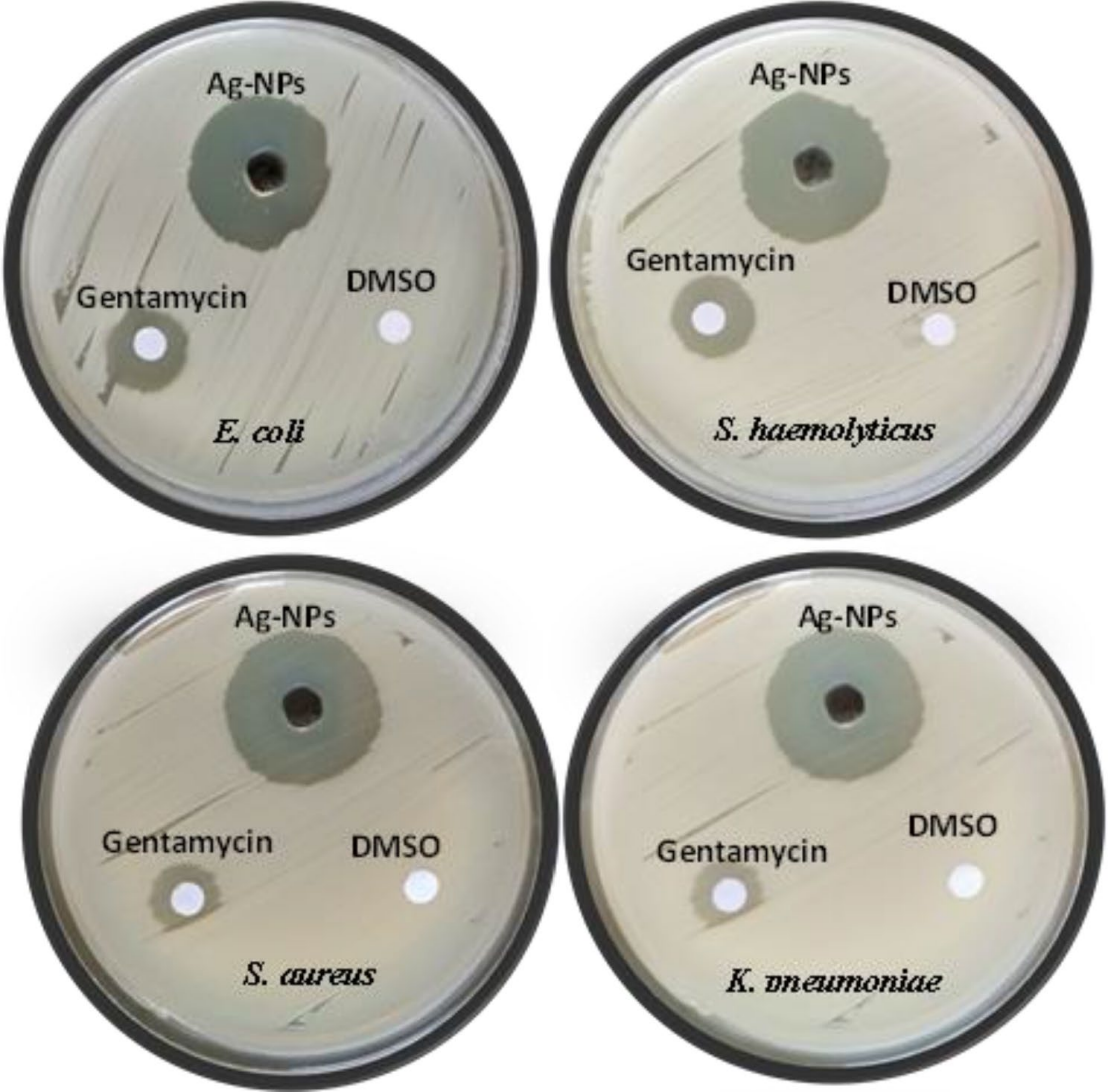
Comparing the antimicrobial action (mm) of the prepared AgNPs versus *E. coli*,* S. haemolyticus*,* S.aureus* and *K.pnumonia*, relative to gentamycin as standard antibiotic and DMSO as negative control

The antimicrobial activity of the biosynthesized AgNPs showed a notable enhancement (*P* ≤ 0.05) compared to gentamicin, the reference antibiotic, against both Gram-positive and Gram-negative bacterial strains. The AgNPs demonstrated potent antibacterial effects against the Gram-positive bacteria *S. aureus* and *S. haemolyticus*, as well as the Gram-negative bacteria *E. coli* and *K. pneumoniae* (Table 2; Fig. 5). Statistical analysis using one-way ANOVA showed highly significant differences among the treatment groups (*P* < 0.0001). The F-values for *S. aureus*,* S. haemolyticus*,* E. coli*, and *K. pneumoniae* were 1721.18, 7265.60, 16042.33, and 5051.18, respectively, indicating a significant impact of AgNPs treatment on bacterial inhibition.

**Table 2:** Antibacterial impact (mm) of the prepared AgNPs and gentamicin various different bacteria (Data are represented as means ± SD; * *P* ≤ 0.05 refer to significant difference relative to standard antibiotic)

No.	Isolate name	Diameter of inhibition zone (mm) by applying 100 µl of sample		
		Control		AgNPs
		Gentamycin	D. water	
1	*Staphylococcus aureus* (ATCC25923)	14.0 ± 0.2	0	28.0 ± 1.0*
2	*Staphylococcus haemolyticus* (ATCC29968)	16.1 ± 0.6	0	27.0 ± 0.4*
3	*Escherichia coli* (ATCC8739)	14.2 ± 0.3	0	28.0 ± 0.3*
4	*Klebsiella pneumonia* (ATCC2146)	15.0 ± 0.4	0	30.0 ± 0.6*

The MIC and MLC values for *S. aureus* were 31.25 ± 0.4 µg/ml and 62.5 ± 1.0 µg/ml, respectively, while those for *S. haemolyticus* were 31.25 ± 0.6 µg/ml and 62.5 ± 0.2 µg/ml, respectively. Additionally, the MIC and MLC values for *E. coli* were 62.5 ± 0.3 µg/ml and 125.0 ± 0.3 µg/ml, respectively, whereas for *K. pneumoniae*, the MIC and MLC values were 62.5 ± 0.8 µg/ml and 125.0 ± 0.2 µg/ml, respectively (Table 3). Statistical analysis using one-way ANOVA showed a significant difference in antibacterial activity among the tested bacterial strains (*P* < 0.05). The F-values for the MIC and MLC were calculated to be 18.24 and 22.76, respectively, indicating a statistically significant difference in the susceptibility of Gram-positive and Gram-negative bacteria to AgNPs treatment. These results suggest that the biosynthesized AgNPs had a more pronounced antibacterial effect on Gram-positive bacteria compared to Gram-negative bacteria, as evidenced by the lower MIC and MLC values.

**Table 3:** Determination of minimum inhibitory (MICs) and minimum lethal (MLCs) concentrations against bacterial strains (µg/ml) (Data are represented as means ± SD)

No.	Isolate name	Minimum inhibitory (MICs) and Minimum Lethal (MLCs) concentrations against bacterial strains(µg/ml)	
		AgNPs	
		MIC	MLC
1	*Staphylococcus aureus* (ATCC25923)	31.25 ± 0.4	62.5 ± 1.0
2	*Staphylococcus haemolyticus* (ATCC29968)	31.25 ± 0.6	62.5 ± 0.2
3	*Escherichia coli* (ATCC8739)	62.5 ± 0.3	125 ± 0.3
4	*Klebsiella pneumonia* (ATCC2146)	62.5 ± 0.8	125 ± 0.2

### Antioxidant action of the prepared AgNPs

**Fig. 6:**
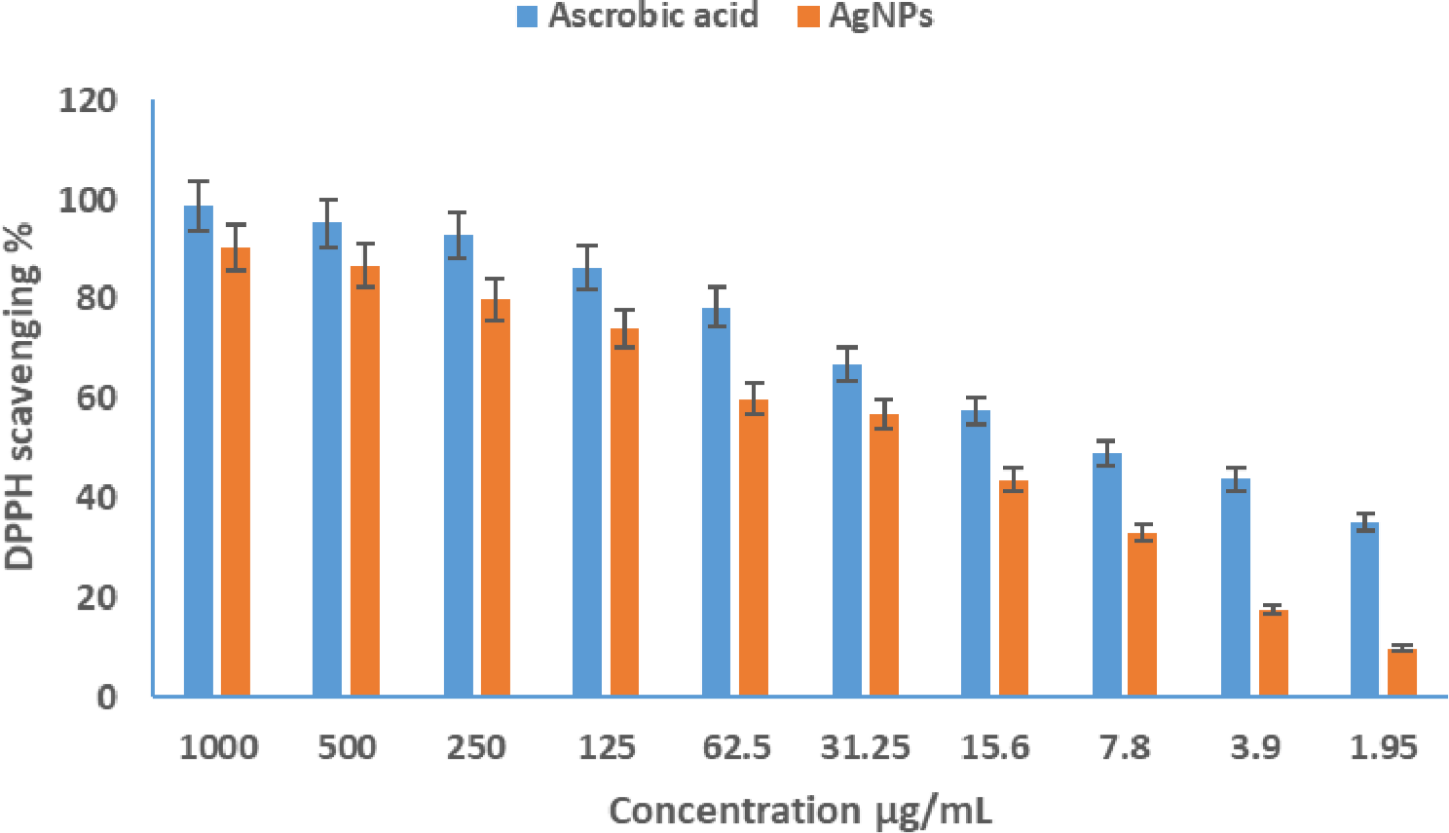
Determination of the antioxidant impact of the prepared AgNPs using the DPPH assay at concentrations ranging from 1000 to 1.95 µg/mL (Data are represented as means ± SD)

In this study, the antioxidant activity of the biosynthesized AgNPs was assessed and compared with ascorbic acid, which served as the reference standard. The IC₅₀ value for ascorbic acid was found to be 2.8 ± 0.1 µg/ml, indicating its potent antioxidant capacity. In contrast, the IC₅₀ value for the synthesized AgNPs was 4.94 µg/ml, reflecting a moderate yet significant antioxidant activity (Fig. 6). Statistical analysis, performed using one-way ANOVA, resulted in an F-value of 0.00047 and a p-value of 0.9995, which indicates no statistically significant difference among the replicate measurements. While the AgNPs exhibited lower antioxidant activity compared to ascorbic acid, they still showed notable free radical scavenging ability. The difference in IC₅₀ values indicates that a higher concentration of AgNPs is required to achieve 50% radical inhibition compared to ascorbic acid. This antioxidant potential may be attributed to the surface interactions between AgNPs and ROS, which facilitate electron transfer and neutralization of free radicals. These findings highlight the capability of the biosynthesized AgNPs as potential antioxidant agents, which could be further explored for biomedical and pharmaceutical applications.

### Anti-inflammatory role of the synthesized AgNPs

**Fig. 7:**
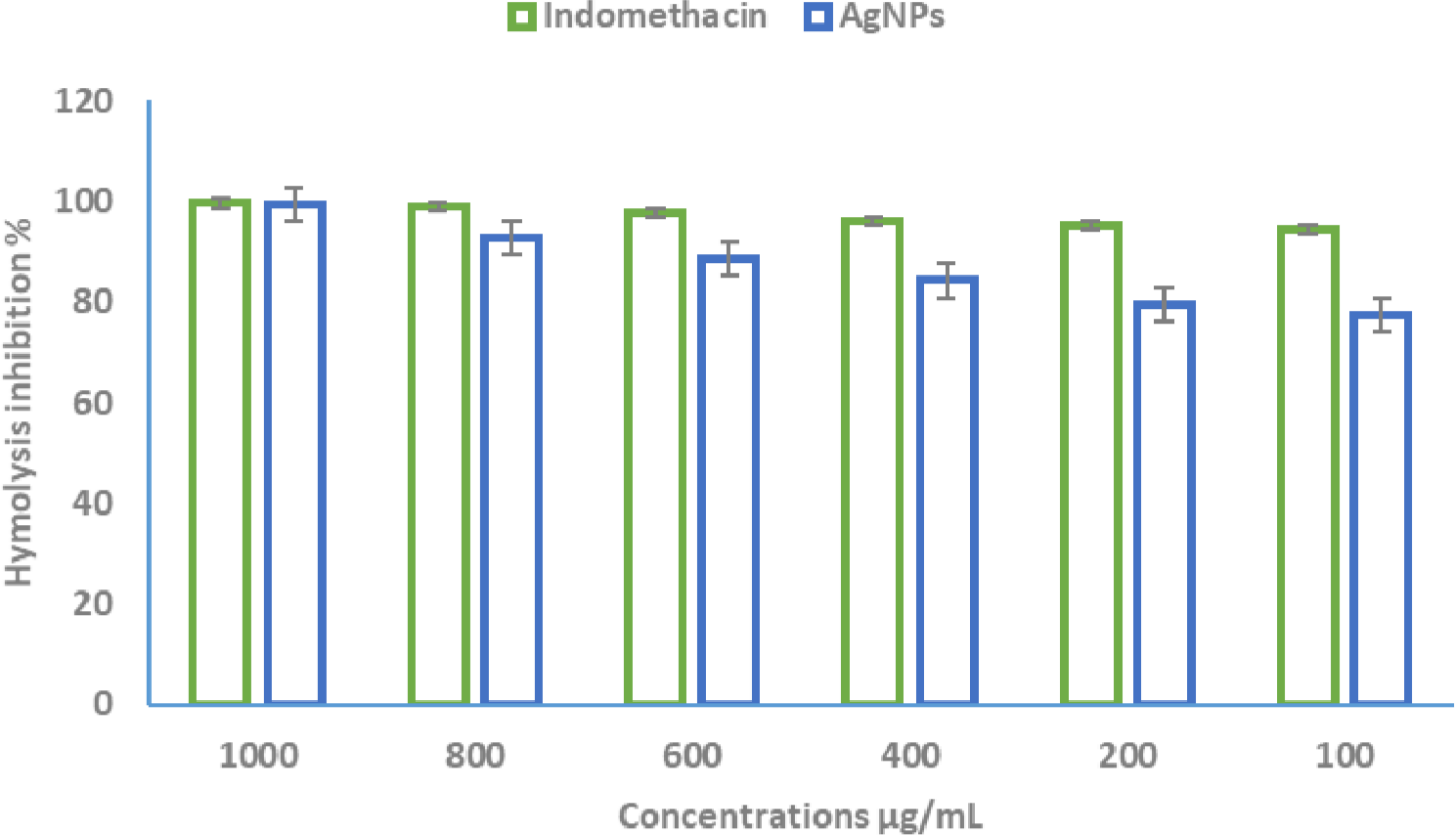
Evaluation of the anti-inflammatory activity of the synthesized AgNPs using the membrane stabilization technique at concentrations ranging from 1000 to 100 µg/mL (Data are expressed as mean ± SD)

The rupture of erythrocytes was examined using various concentrations of the biosynthesized AgNPs and compared to the positive control, indomethacin. The IC₅₀ value of indomethacin was determined to be 3.5 ± 0.1 µg/ml, demonstrating its potent anti-inflammatory effect. Meanwhile, the IC₅₀ value of the biosynthesized AgNPs was 7.14 µg/ml, indicating a moderate but significant anti-inflammatory potential (Fig. 7). The results suggest that while the AgNPs exhibited lower anti-inflammatory activity compared to indomethacin, they still demonstrated a considerable ability to stabilize erythrocyte membranes and inhibit hemolysis. The higher IC₅₀ value of AgNPs suggests that a relatively higher concentration is required to achieve 50% inhibition of membrane rupture compared to the standard drug. This protective effect against erythrocyte lysis could be attributed to the interaction of AgNPs with cellular membranes, leading to reduced oxidative stress and inhibition of inflammatory mediators. Moreover, the dose-dependent anti-inflammatory activity of AgNPs suggests their potential role as an alternative anti-inflammatory agent.

### Cytotoxic assay of the biosynthesized AgNPs

**Fig. 8:**
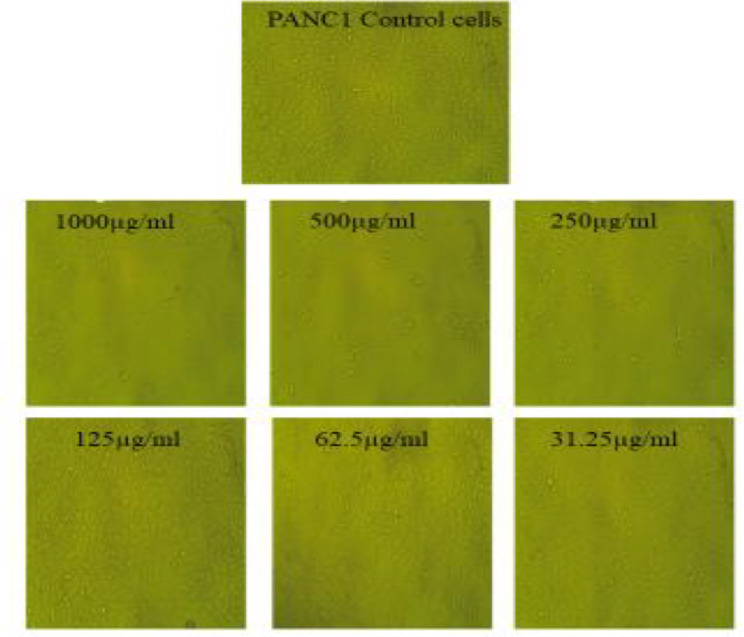
Anticancer impact of various levels of AgNPs ranging from 1000 µg/ml to 31.25 µg/ml towards PANC1 cells using inverted microscope (X = 400)

**Fig. 9:**
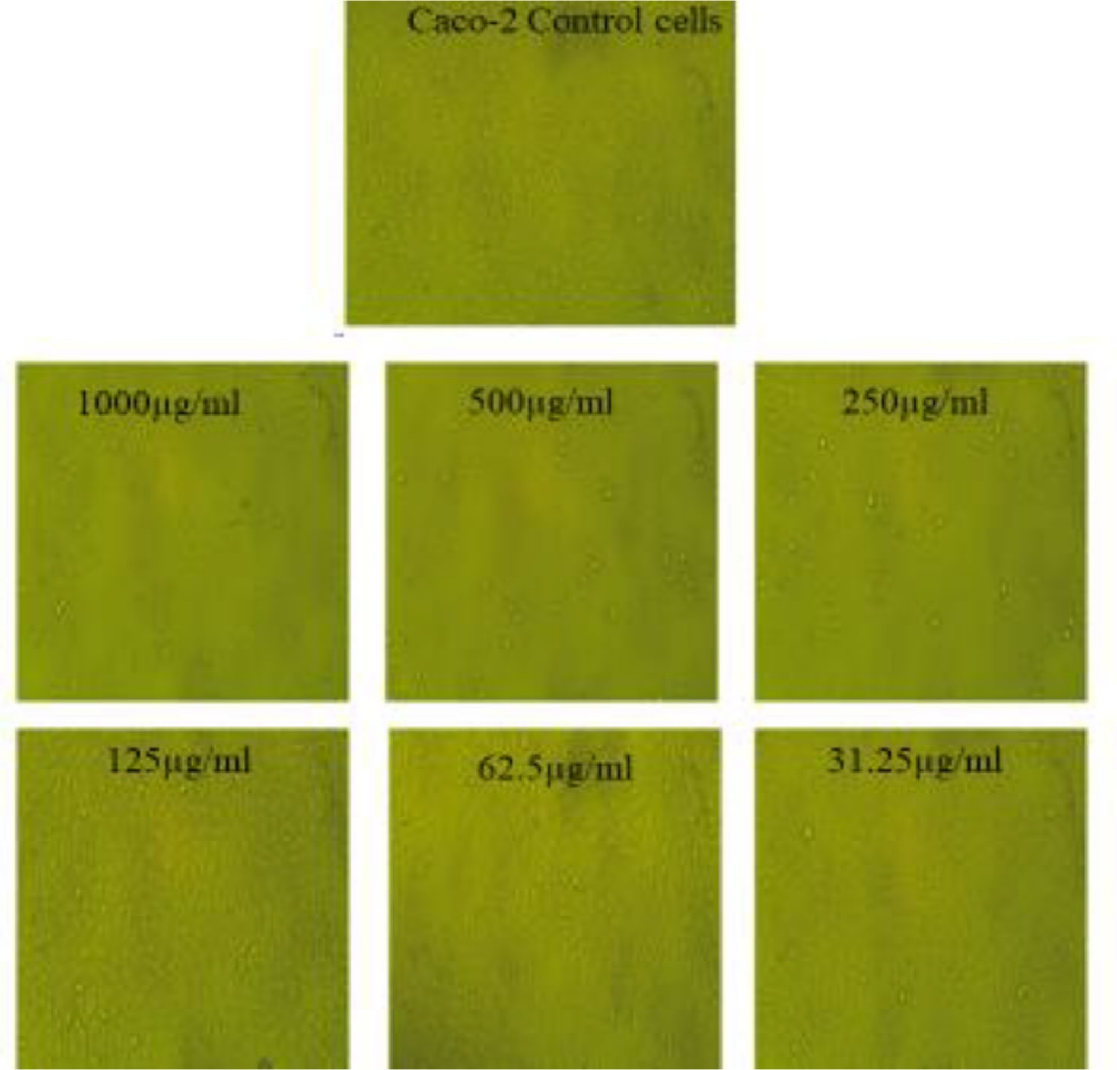
Microscopic examination of Caco-2 cells upon using various levels of the prepared AgNPs ranging from 1000 µg/ml to 31.25 µg/ml (X = 400)

**Fig. 10:**
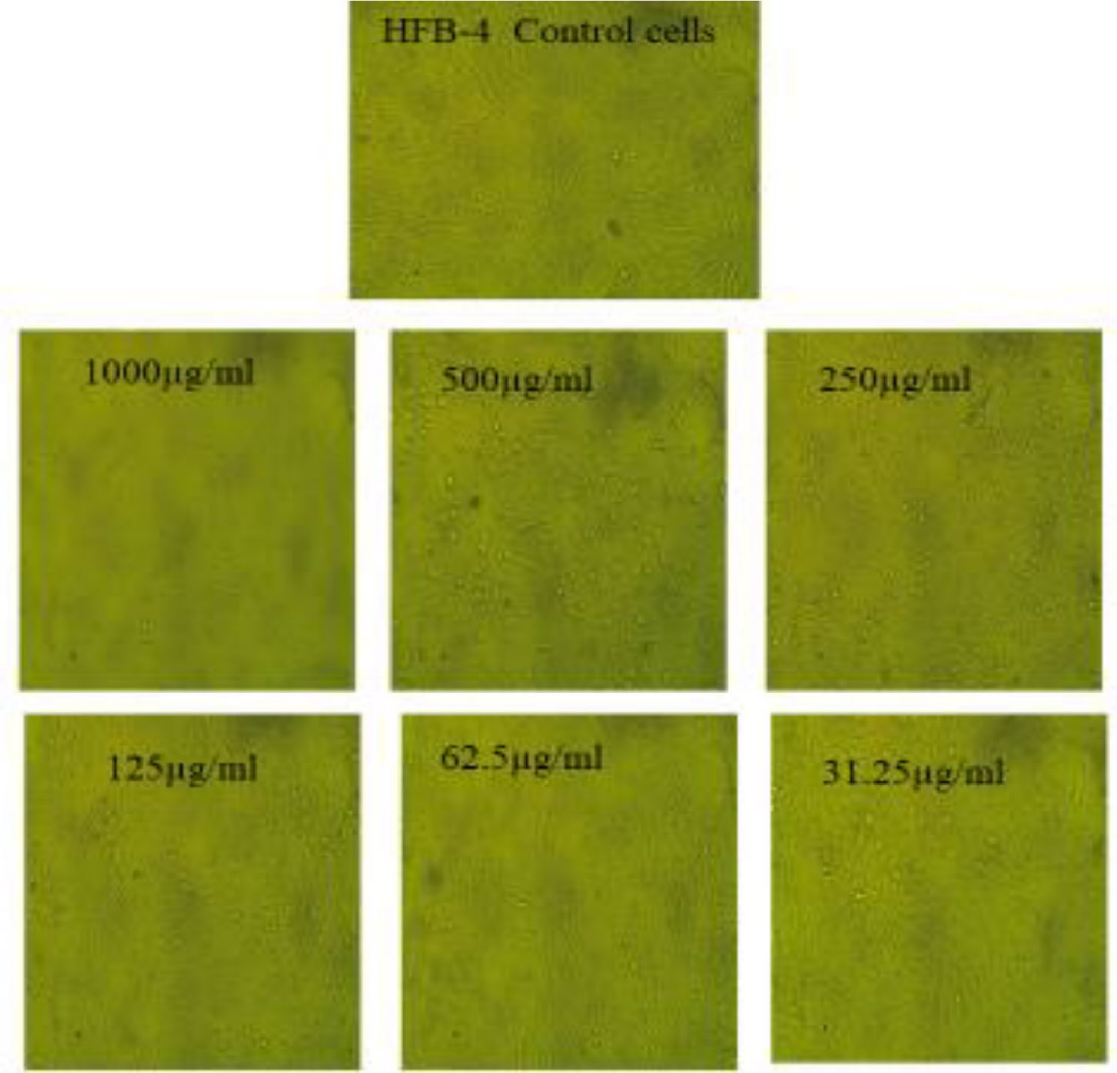
Microscopic examination of HFB-4 cells upon using various levels of the prepared AgNPs ranging from 1000 µg/ml to 31.25 µg/ml (X = 400)

The cytotoxic potential of the biosynthesized AgNPs was evaluated using the MTT assay across a concentration range of 1000–31.25 µg/ml. The results demonstrated a significant cytotoxic effect of AgNPs against Caco-2 and PANC-1 cell lines, with IC₅₀ values of 177.24 ± 2.01 µg/ml and 208.15 ± 2.79 µg/ml, respectively (Figs. 8 and 9; Tables 4 and 5). This suggests that AgNPs exhibit a dose-dependent cytotoxic response, effectively inhibiting cancer cell proliferation. In contrast, the AgNPs displayed minimal toxicity against normal human fibroblast cells (HFB-4), with an IC₅₀ value of 582.33 ± 6.37 µg/ml, indicating their biocompatibility and selective action towards cancerous cells (Fig. 10; Table 6). The significantly higher IC₅₀ for HFB-4 cells suggests that AgNPs exert preferential toxicity toward malignant cells while sparing normal cells, which is a crucial aspect of potential therapeutic applications.

**Table 4:** Determination of anticancer impact of the prepared AgNPs upon using different levels ranging from (1000–31.25 µg/ml) towards PANC1 (Data are represented as means ± SD)

ID	µg/ml	O.D			Mean O.D	±SE	Viability %	Toxicity %	IC_50_ ± SD
PANC1	--------	0.768	0.77	0.772	0.77	0.001155	100	0	µg
Ag	1000	0.052	0.044	0.041	0.045667	0.003283	5.93075931	94.0696407	209.15 ± 2.99
	500	0.056	0.066	0.051	0.057667	0.00441	7.48917489	92.5102251	
	250	0.247	0.281	0.269	0.265667	0.009955	34.502645	65.497355	
	125	0.642	0.619	0.638	0.633	0.007095	82.2079221	17.7920779	
	62.5	0.76	0.754	0.768	0.760667	0.004055	98.7877879	1.21211212	
	31.25	0.766	0.771	0.768	0.768333	0.001453	99.7834978	0.21640216	

**Table 5:** Detection of antitumor action of the synthesized AgNPs upon using various concentrations ranging from (1000–31.25 µg/ml) towards Caco-2 cells (Outcomes are tabulated as means ± SD)

ID	µg/ml	O.D			Mean O.D	±SE	Viability %	Toxicity %	IC_50_ ± SD
Caco2	--------	0.636	0.63	0.627	0.631	0.002646	100	0	µg/ml
(AgNP)	1000	0.078	0.083	0.058	0.073	0.007638	11.5689819	88.4310681	178.24 ± 2.11
	500	0.066	0.068	0.072	0.068667	0.001764	10.8829757	89.1178043	
	250	0.133	0.126	0.111	0.123333	0.006489	19.5469466	80.4540534	
	125	0.438	0.462	0.442	0.447333	0.007424	70.8976281	29.1073719	
	62.5	0.632	0.63	0.624	0.628667	0.002404	99.6302169	0.36978413	
	31.25	0.625	0.634	0.631	0.63	0.002646	99.8415139	0.15878605	

**Table 6:** Detection of cytotoxic role of the synthesized AgNPs upon using different levels ranging from (1000–31.25 µg/ml) towards HFB-4 cells (Data are represented as means ± SD)

ID	µg/ml	O.D			Mean O.D	±SE	Viability %	Toxicity %	IC_50_ ± SD
HFB4	-------	0.839	0.822	0.823	0.828	0.00550	100	0	µg/ml
(AgNPs)	1000	0.038	0.023	0.026	0.029	0.00458	3.502415459	96.4978454	583.34 ± 6.38
	500	0.376	0.411	0.427	0.404667	0.015059	48.87278583	51.1271417	
	250	0.817	0.795	0.803	0.805	0.00642	97.2222222	2.77777778	
	125	0.835	0.821	0.825	0.827	0.00416	99.8792705	0.12077947	
	62.5	0.831	0.823	0.827	0.827	0.00230	99.8792705	0.12077947	
	31.25	0.828	0.822	0.829	0.826333	0.00218	99.7981176	0.20128845	

These findings highlight the promising anticancer properties of AgNPs, which may be attributed to their ability to induce oxidative stress, disrupt mitochondrial function, and trigger apoptosis in cancer cells. The selectivity of AgNPs towards cancer cells over normal cells supports their potential as a safer alternative in cytotoxic treatment.

## Discussion

In recent years, nanotechnology has emerged as a transformative force across various fields, particularly in biomedical and engineering applications, owing to its efficiency, eco-friendliness, safety, and cost-effectiveness [37]. Among the different approaches to nanoparticle synthesis, biologically derived nanoparticles have garnered increasing interest as sustainable alternatives to chemically synthesized counterparts [38]. Numerous studies have explored green synthesis methods for AgNPs using diverse biological materials, including seaweeds, insects, and plant extracts [39–41]. However, the potential of silk derived from *Bombyx mori* a readily available, protein- and fiber rich resource remains largely underexplored for nanoparticle fabrication. In this work silk was processed to AgNPs were prepared using silk from *Bombyx mori.* The water-miscible organic solvent procedure, which allows for smaller sizes than other techniques such as separating phases in salt and freezing, was used to the synthesis of silk fibroin NPs [42, 43]. Acetone was utilized as the RSF solvent to manufacture the NPs as previously reported [44]. The AgNPs biosynthesized using *Bombyx mori* silk extract exhibit several advantages compared to those synthesized using plant extracts in terms of yield, stability, and bioactivity. The controlled release of reducing and stabilizing biomolecules from silk fibroin and sericin results in a higher and more consistent nanoparticle yield, whereas plant-derived AgNPs may exhibit variability due to differences in phytochemical composition [45, 46]. In terms of stability, the presence of protein-based capping agents in silk extract provides enhanced colloidal stability, reducing aggregation and extending shelf life [47], whereas plant-derived AgNPs often rely on polyphenols and flavonoids, which may degrade over time, leading to particle instability [48]. Moreover, the protein matrix in silk enhances the biocompatibility of AgNPs, making them more suitable for in vivo applications and potential clinical translation [49]. The scalability of silk-based synthesis further supports its feasibility for large-scale production, making it a viable alternative for industrial and pharmaceutical applications.

The produced NPs’ XRD patterns were identified in order to evaluate the molecular interactions [50]. The diffraction pattern obtained confirms that the AgNPs exhibit a face-centered cubic (fcc) structure, affirming their crystalline nature [51]. The diffraction peaks observed at 2θ values of 38.06, 44.24, 64.45, and 77.61 correspond to the (1 1 1), (2 0 0), (2 2 0), and (3 1 1) planes, respectively, which further supports the fcc structure of the flaxseed-mediated silver nanoparticles, as previously reported (JCPDS: file No-087–0720).

Surface plasmon resonance excitation is a well-established phenomenon associated with nanoparticle formation [52]. The successful biosynthesis of AgNPs in this study was confirmed by the distinct absorbance peak at 420 nm, consistent with previously reported SPR values for silver nanoparticles, such as those observed by Bahrami-Teimoori et al. [53]. This finding underscores the efficiency of silk-derived compounds in facilitating the reduction of metal salts into metallic nanoparticles, highlighting their potential as sustainable reducing agents. Transmission electron microscopy analysis revealed that the AgNPs were predominantly circular, with a size distribution ranging from 10 to 30 nm. The role of bio-surfactants in nanoparticle synthesis, particularly their influence on pH and particle uniformity, has been highlighted in previous studies. Reddy et al. [54] reported that adjusting the pH to 9 led to more homogeneous nanoparticles, although some variations in size were still observed, aligning with findings by Ahmed et al. [55].

The FTIR spectra reveal significant differences between *Bombyx mori* silk and the AgNPs biosynthesized using the silk extract, confirming structural and functional modifications during nanoparticle formation. The broad peak around 3344–3348 cm⁻¹ in both spectra corresponds to O-H and N-H stretching vibrations, indicating the presence of hydroxyl and amine groups [56]. However, the slight shift in this peak in AgNPs suggests possible hydrogen bonding interactions between silk biomolecules and the nanoparticles, playing a role in stabilization [57].

The peaks at 2959, 2873, and 2833 cm⁻¹ in *Bombyx mori* silk correspond to aliphatic C-H stretching vibrations, which are slightly modified in AgNPs (2986 cm⁻¹), indicating structural rearrangements during synthesis. The presence of a peak at 2164 cm⁻¹ in APNPs, which is absent in the silk spectrum, suggests new chemical interactions, possibly related to C ≡ C or C ≡ N stretching vibrations [58]. This could be attributed to bioorganic compounds in the silk extract interacting with nanoparticle surfaces. In the fingerprint region, major shifts in the amide bands (1648, 1464, and 1378 cm⁻¹ in silk) toward 1598 and 1374 cm⁻¹ in AgNPs indicate strong interactions between protein functional groups and the nanoparticle surface [59]. The new absorption peaks at 1045, 594, 531, and 449 cm⁻¹ in the AgNP spectrum, which are absent in pure silk, correspond to metal-oxygen (M-O) vibrations, confirming the formation of metal-based nanoparticles [57]. The emergence of these peaks is direct evidence of successful nanoparticle synthesis, with biomolecules from *Bombyx mori* silk acting as reducing and stabilizing agents in the process. Similar observations have been made by Soman and Ray [60], as well as Selvakumar et al. [61], who demonstrated the efficacy of *Ziziphus oenoplia* (L.) and *Acalypha hispida* leaf extracts in acting as natural stabilizers and reducing agents. These findings collectively emphasize the versatility of biological materials in green nanoparticle synthesis and open avenues for further exploration of silk-based nanomaterials in biomedical and industrial applications.

**Scheme 1:**
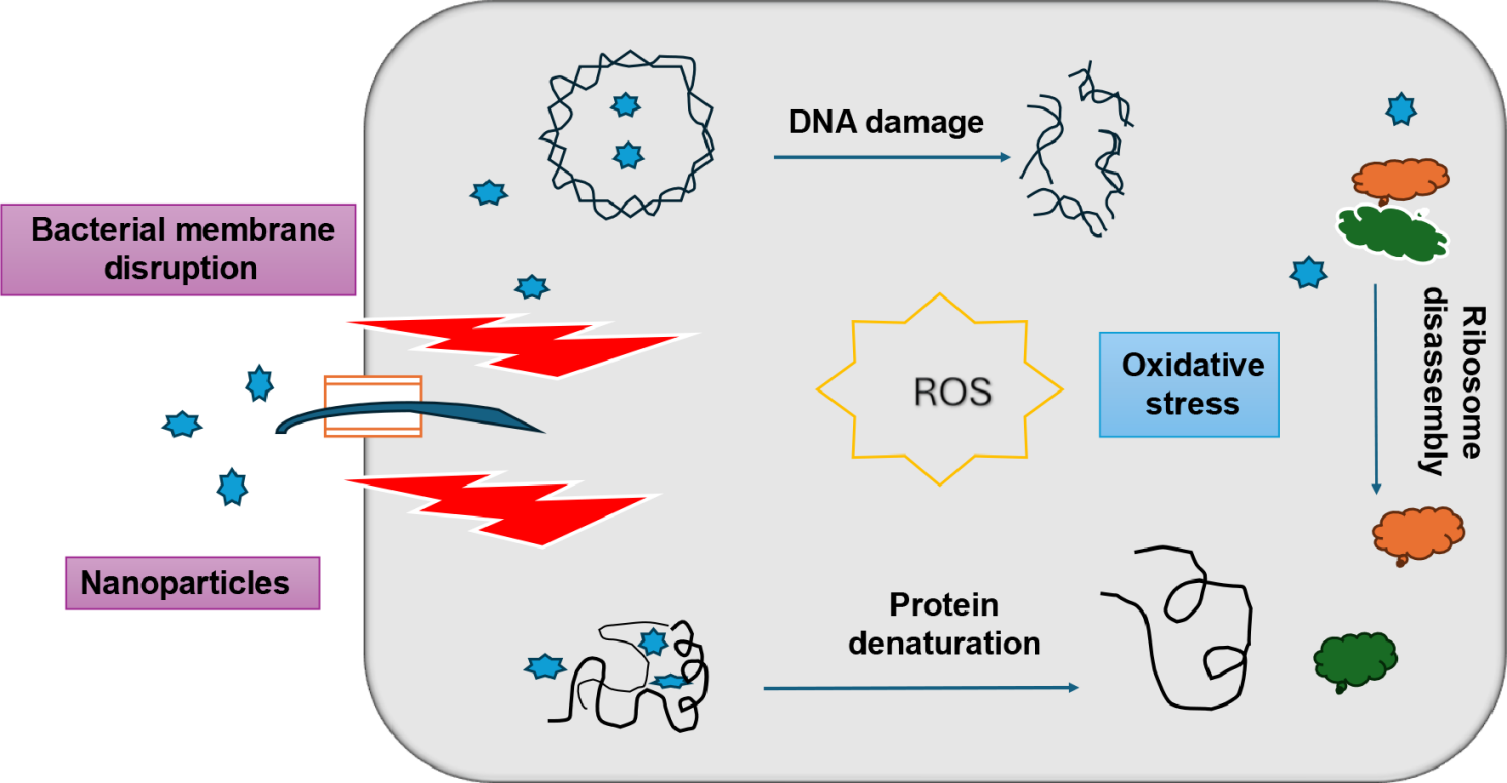
Possible mechanism of action of AgNPs for antimicrobial activity

The biosynthesized AgNPs exhibited strong antibacterial activity against *S. aureus*, *S. haemolyticus*, *E. coli*, and *K. pneumoniae*, indicating their potential as an alternative to conventional antibiotics. Their nanoscale size enables them to adhere to bacterial membranes, disrupt cell integrity, and increase permeability, ultimately leading to bacterial death as illustrated in Scheme 1 [62]. Several studies have reported similar antibacterial effects of AgNPs. For instance, Hosnedlova et al. [63] demonstrated that biologically synthesized AgNPs exhibited strong antimicrobial activity against *S. aureus* and *E. coli*, with significant membrane damage observed under electron microscopy. Their findings align with the proposed mechanism in this study, where AgNPs disrupt bacterial cell walls and interfere with metabolic processes. Additionally, a study by Abbigeri et al. [64] reported that AgNPs induce oxidative stress by generating ROS, leading to DNA fragmentation and protein denaturation in *K. pneumoniae*, further supporting the hypothesis that AgNPs exert their bactericidal effect through multiple pathways. The efficacy of AgNPs can vary based on size, concentration, and bacterial strain type. Studies suggest that Gram-negative bacteria like *E. coli* and *K. pneumoniae* may be more susceptible due to their thinner peptidoglycan layer and porin channels, which facilitate nanoparticle penetration [65]. However, variations in nanoparticle synthesis methods, stabilizing agents, and bacterial resistance mechanisms could influence the overall antimicrobial activity [66]. While these findings highlight the promising potential of AgNPs in combating bacterial infections, further investigations are needed to assess their long-term stability, cytotoxicity, and clinical applicability.

The utilization of biosynthesized AgNPs as naturally occurring antioxidants was recommended by the study’s findings. Proteins and other biomolecules in the AgNPs solution donate hydrogen molecules, which is the primary cause of the AgNPs’ ability to scavenge free radicals [41]. The occurrence of bioreductant molecules on the surface of AgNPs increases the surface area available for antioxidant capacity, which in turn causes radical scavenging action [67]. As a result, these AgNPs can be used to balance the levels of ROS and as a natural antioxidant in pro-oxidants. AgNPs produced with *Catharanthus roseus* shown the capacity to neutralize radicals in a prior work, preventing degenerative illnesses and harm to human cells [68].

Hemolysis is often used as a key indicator of erythrocyte membrane damage caused by free radicals [69]. In contrast, antioxidant and anti-inflammatory compounds are known for their ability to neutralize free radicals, thereby protecting cells from hemolysis induced by oxidative stress. Additionally, the synthesized nanoparticles demonstrated a promising anti-inflammatory effect. This aligns with findings from other researchers [70, 71], who observed similar anti-inflammatory effects of green-synthesized silver nanoparticles.

The substance’s chemical structure, level, and interactions with target species can all affect its impact. AgNPs are suggested as anticancer agents due to their toxic effects and security depending on their tiny dimensions and extensive surface area for contact with biological systems [72]. In the present work the prepared AgNPs showed anticancer potential towards Caco-2 and PANC1 cells as cancer cells with minimal toxic impact towards HFB4 as normal cells. According to recent studies, a number of silver compounds affect cancer cells in different ways [73]. Silver exhibits low toxicity but limited absorption due to the body’s efficient detoxification system. AgNPs offer a practical solution to this issue. Specifically, mechanisms like endocytosis enable cells to take up AgNPs, where they release Ag + ions, the reactive form of silver, at targeted sites [74].

The biological effects of the biosynthesized AgNPs, particularly their anticancer activity, can be attributed to several possible mechanisms, including ROS generation, apoptosis induction, mitochondrial dysfunction, and DNA damage [75]. AgNPs are known to induce oxidative stress by generating ROS, which disrupts cellular homeostasis, leading to lipid peroxidation, protein oxidation, and DNA fragmentation [76]. This oxidative stress can activate intrinsic apoptotic pathways by triggering mitochondrial membrane depolarization, leading to cytochrome c release and subsequent activation of caspases, particularly caspase-3 and caspase-9, which play a crucial role in programmed cell death [77]. Moreover, AgNPs have been reported to interact with cellular proteins, impairing cytoskeletal integrity and disrupting normal cell division. The selective cytotoxicity observed in cancer cells compared to normal fibroblasts suggests that AgNPs may preferentially target rapidly dividing cells, possibly due to differences in membrane composition and nanoparticle uptake [78]. Furthermore, AgNPs can induce autophagy, which may contribute to cancer cell death through self-digestion of essential cellular components. These combined mechanisms highlight the potential of AgNPs as effective anticancer agents, warranting further studies to elucidate their precise molecular interactions and optimize their therapeutic applications [79].

## Conclusion

The silk produced by *Bombyx mori* was successfully used to biosynthesize AgNPs (5–25 nm) with promising multi-therapeutic potential. The AgNPs exhibited potent antimicrobial activity against *S. aureus*,* S. haemolyticus*,* E. coli*, and *K. pneumoniae*, surpassing gentamicin. Their antioxidant capacity (IC₅₀ = 4.94 µg/ml) was comparable to ascorbic acid, while their anti-inflammatory effect (IC₅₀ = 7.14 µg/ml) approached that of indomethacin. Additionally, AgNPs demonstrated selective cytotoxicity against Caco-2 and PANC-1 cancer cells (IC₅₀ = 177.24 and 208.15 µg/ml) while showing minimal toxicity towards normal fibroblasts (IC₅₀ = 582.33 µg/ml). Given these promising in vitro results, future studies should focus on in vivo validation, exploring formulation strategies for enhanced bioavailability, and assessing clinical translation potential for biomedical applications.

## Data Availability

All the data in the article is presented in the form of images, tables and graphs.
